# Supplementary material for: Ablation of SMUG1 Reduces Cell Viability and Increases UVC-Mediated Apoptosis in Hepatocarcinoma HepG2 Cells
Source: Genes (Basel). 2021 Jan 30;12(2):201. doi: 10.3390/genes12020201 (PMC7911780; doi:10.3390/genes12020201)
Supplement: Supplementary file 1 [file genes-12-00201-s001.zip › genes-1071656 supplementary/Supplementary File 1.docx]

**Supplementary Materials and Methods**

***1. MTT assay***

Cell proliferation rate was measured by MTT (3-(f,5-dimethyl-thiazol-2-ul)-2,5-diphenyl-2H-tetrazolium bromide) assay. Approximately 5 x 10^3^ cells were seeded in 96-well culture plate and allowed to attach for 24 hs. After incubation for 12, 24, 36 and 48 hs, diluted MTT solution (500 ug/ml) added to 100 ul of medium in each well. Cells were incubated for additional 2 hs allowing MTT metabolism in the dark condition. The medium was then aspirated, 200 ul of dimethyl sulfoxide (DMSO, Duchefa) was added into the wells to dissolve the purple formazan crystals. The absorbance of the plate was measured at 570 nm using a plate reader (Multiskan™ FC Microplate Photometer, Thermo fisher scientific).

***2. Immunocytochemistry***

HepG2 WT and SMUG1 KO cells were grown on sterile glass coverslips in 60 mm dishes. The cells were washed with 1x PBS and fixed for 1 h with 4% paraformaldehyde. The cells were permeabilized with 0.3% Triton X-100 in PBS for 30 min, and incubated with primary antibodies with 1% BSA in TBST for 3 to 5 h at room temperature. After incubation, samples were washed with 1% BSA in PBST, and then reacted with the appropriate secondary antibodies for 1 h. 3 ug/ml of DAPI (Sigma) was used for nuclear staining. Fluorescence images were obtained with Zeiss Axio Observer Z1 LSM 700 confocal microscope (Carl zeiss, Oberkochen, Germany).

***3. Immunoblot***

The collected cells were lysed in a buffer containing 1% Triton X-100, 150 mM NaCl, 50 mM Tris-HCl, pH 7.5, 0.1% sodium dodecyl sulfate (SDS), 1% Nonidet P-40, and 1 mM PMSF by vortexing. After centrifugation, the protein concentration of the supernatant was measured by the colorimetric assay (Biorad, #5000116). The protein samples were electrophoresed on a 10% SDS-PAGE and transferred to a nitrocellulose membrane (Protran™; Whatman, Maidstone, UK). The membrane was blocked with 5% skim milk in a solution of 20 mM Tris–HCl (pH 7.6), 137 mM NaCl and 0.1% Tween-20, and incubated with appropriate dilutions of the primary antibody at room temperature for 3 hrs. Samples were analyzed by Western blotting using the appropriate antibodies to detect protein expression. The list of antibodies was described in Table S1.

Table S1. Antibody list

| **Antibody** | **Company** | **#Cat** | **Species** | **Clonality** | **Dilution** | **Applications** |
| --- | --- | --- | --- | --- | --- | --- |
| SMUG1 | Santacruz | sc-98849 | Rabbit | Poly | 1:200  1:1000 | Immunocytochemistry  Immunoblot |
| β-actin | Santacruz | sc-47778 | Rabbit | Poly | 1:1000 | Immunoblot |
| P-histone-H2X(ser139) | Millipore | 05-636 | Mouse | Mono | 1:1000 | Immunoblot |
| P-histone-H2AX(ser139) | Ab cam | Ab2893 | Rabbit | Poly | 1:100 | Immunocytochemistry |
| Cytochrome C | Santacruz | sc-13156 | Mouse | Mono | 1:200  1:1000 | Immunocytochemistry  Immunoblot |
| Cyclin A | Santacruz | Sc-239 | Mouse | Mono | 1:1000 | Immunoblot |
| Cyclin B | Cell signaling | #4138 | Rabbit | Poly | 1:1000 | Immunoblot |
| CDK1 | Abcam | Ab18 | Mouse | Mono | 1:1000 | Immunoblot |
| P53 | Santacruz | sc-126 | Mouse | Mono | 1:1000 | Immunoblot |
| P21 | Santacruz | sc-817 | Mouse | Mono | 1:500 | Immunoblot |

***4. UV light exposure***

UVC irradiation was performed in UVP chamber (UVP; CA, USA) using UVC lamp with four G8T5 bulbs, wavelengths 253.7 nm. HepG2 cells were exposed 75 mJ/cm^2^ UVC. After the UVC irradiation, the cells were returned to 37°C incubator with 5% CO_2_ at humidified atmosphere.

***5. Generation of SMUG1 Knock-down (KD) stable cell line***

To create the shRNA expression vector targeting for human *SMUG1* gene, we designed target DNA oligonucleotides were sub-cloned into pLKO.1-puro (Addgene, Plasmid #8453) lentiviral vector. Then HEK293T cells were co-transfected with the plasmids encoding VSV-G lentiviral vector, packaging plasmid pCDNA3-NLBH and the shRNAs. Two days after transfection, the supernatant that containing the virus particles were collected and used for infection of HEK293T cells in the presence of polybrene (8 mg/ml).

***6. DNA fragmentation assay***

Programmed cell death was measured by apoptotic DNA fragmentation assay. The cells were dissolved in 100 ul of cell-lysis buffer which containing 20 mM EDTA, 50 mM Tris-cl (pH 7.5) and 1% NP-40. The lysates were then centrifuged at 16,000g for 5 min at 4℃. The supernatant was recovered and treated with RNase A for 1 h at 37℃, and followed by the treatment of Proteinase K for 12 hs at 56℃. DNA was precipitated by adding 900 ul of 100% ethanol and 120 ul of 10 M ammonium acetate, and the precipitates were resuspended in D.W. Same amount of 20 μg of gDNA samples were electrophoresed on 1.5% agarose gel for 20 min to visualize the DNA ladder formation.
