# Supplementary figures and images for: Ablation of SMUG1 Reduces Cell Viability and Increases UVC-Mediated Apoptosis in Hepatocarcinoma HepG2 Cells
Source: Genes (Basel). 2021 Jan 30;12(2):201. doi: 10.3390/genes12020201 (PMC7911780; doi:10.3390/genes12020201)

# Supplementary Figure 1

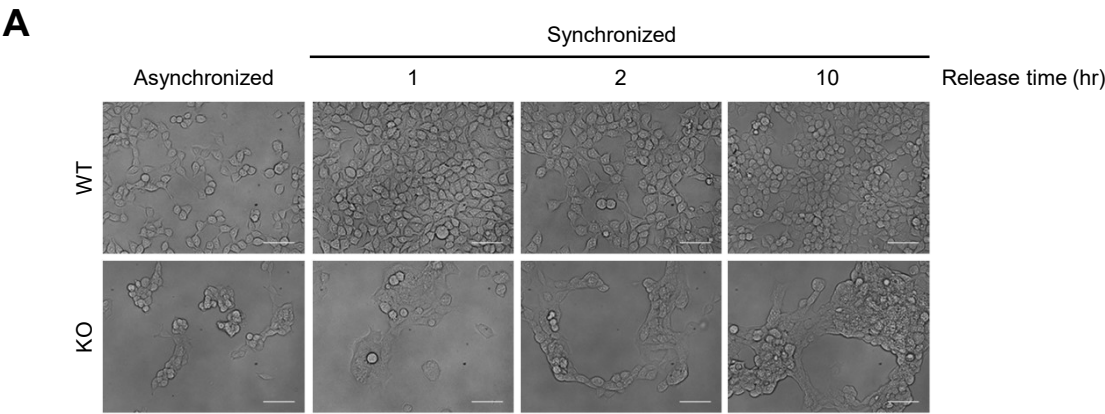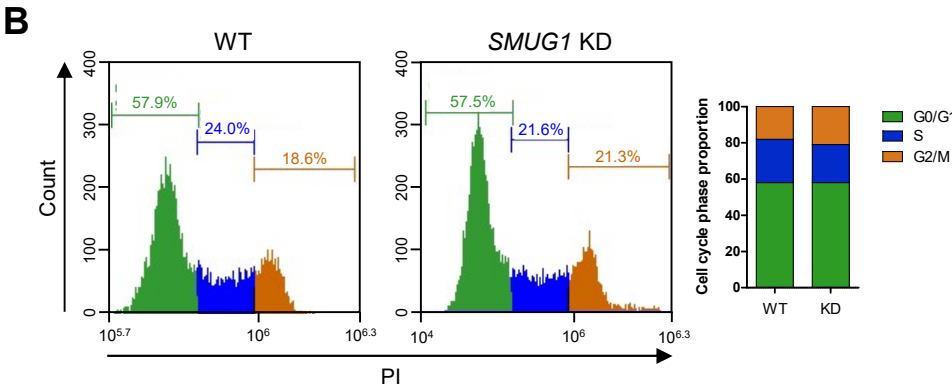

Supplement: Supplementary file 1 [file genes-12-00201-s001.zip › genes-1071656 supplementary/Supplementary Figures.pdf]
